# Pathological Similarity between Pneumonia and Bubonic Plague and Pandemic Influenza

**Published:** 1919

**Authors:** 


**Pathological Similarity between Pneumonia, Bubonic Plague and Pandemic Influenza.** By D. SYMMERS. *The Journal of the American Medical Association*, November 2, 1918.

The changes in the lungs in cases of pandemic influenza are those of a variety of confluent lobular exudative and hemorrhagic pneumonias in which the naked eye and microscopic features bear a remarkable resemblance to the lesions of the pneumonic variety of bubonic plague. Moreover, the similarity of the two diseases is enforced by the clinical features which are alike in many respects, and by the pathology of certain tissues other than the lungs. On the other hand, no B. pestis has ever been found in bacteriological investigations. The naked eye appearance of the lungs themselves, however, are suggestive, and may be grouped as follows :

1. Cases with (*a*) extensive confluent lobular solidification of the lower lobes; (*b*) circumscribed or partially confluent consolidation of the lobules of the upper lobes; (*c*) areas of acute vesicular emphysema.

2. More prolonged cases characterized by complete or almost complete consolidation of all or most of the lobes of both lungs, areas of acute vesicular emphysema occurring as an inconspicuous feature.

3. Those cases in which the pneumonic process is obviously subsiding.

*Circulatory System.* In the prevailing pandemic influenza, clinical indications of circulatory troubles are numerous : the nasopharynx is deeply congested; duskiness and cyanosis are common; the blood pressure is often very low and the pulse slow. The anatomic and histologic changes in the circulatory apparatus are of importance as indicating the necessity for stimulation from the instant that diagnosis of influenza is made.

Moreover, the distribution of pathologic changes in the lungs is such as to suggest that the infection is introduced by way of the respiratory tract; every case of pandemic influenza should be regarded from the outset as pneumonic, and so treated.

*Kidneys.* Microscopic examination of the kidneys reveals diffuse congestion of the capillary network, together with cloudy swelling of the tubular epithelium.

*Brain.* In cases attended by delirium, the meninges are richly infiltrated by serous fluid and the capillaries injected. Microscopic examination shows hyperemia. In some eases, purulent meningitis of pneumococcal origin has been observed.

Slight jaundice has occurred in some cases.

*Spleen.* In 7 of the 20 cases observed, the spleen was normal in size; in 9 it was slightly increased; and in the remaining 4 distinctly enlarged.

